# Extra-cell cycle regulatory functions of cyclin-dependent kinases (CDK) and CDK inhibitor proteins contribute to brain development and neurological disorders

**DOI:** 10.1111/gtc.12029

**Published:** 2013-01-07

**Authors:** Takeshi Kawauchi, Mima Shikanai, Yoichi Kosodo

**Affiliations:** 1 Precursory Research for Embryonic Science and Technology (PRESTO), Japan Science and Technology Agency (JST)Saitama, 332-0012, Japan; 2 Department of Physiology, Keio University School of Medicine35 Shinanomachi, Shinjuku-ku, Tokyo, 160-8582, Japan; 3 Department of Anatomy, Keio University School of Medicine35 Shinanomachi, Shinjuku-ku, Tokyo, 160-8582, Japan; 4 Department of Anatomy, Kawasaki Medical School577 Matsushima, Kurashiki, 701-0192, Japan

## Abstract

In developing brains, neural progenitors exhibit cell cycle-dependent nuclear movement within the ventricular zone [interkinetic nuclear migration (INM)] and actively proliferate to produce daughter progenitors and/or neurons, whereas newly generated neurons exit from the cell cycle and begin pial surface-directed migration and maturation. Dysregulation of the balance between the proliferation and the cell cycle exit in neural progenitors is one of the major causes of microcephaly (small brain). Recent studies indicate that cell cycle machinery influences not only the proliferation but also INM in neural progenitors. Furthermore, several cell cycle-related proteins, including p27^kip1^, p57^kip2^, Cdk5, and Rb, regulate the migration of neurons in the postmitotic state, suggesting that the growth arrest confers dual functions on cell cycle regulators. Consistently, several types of microcephaly occur in conjunction with neuronal migration disorders, such as periventricular heterotopia and lissencephaly. However, cell cycle re-entry by disturbance of growth arrest in mature neurons is thought to trigger neuronal cell death in Alzheimer's disease. In this review, we introduce the cell cycle protein-mediated regulation of two types of nuclear movement, INM and neuronal migration, during cerebral cortical development, and discuss the roles of growth arrest in cortical development and neurological disorders.

## Introduction

The balance between the proliferation and differentiation of progenitors determines the size of many organs, including the brain. The timing of the cell cycle exit of neural progenitors is important for the brain morphology and functions, as the defects result in several neurological disorders, including microcephaly (small brain) (Mochida & Walsh 2004; Bond & Woods 2006; Lizarraga *et al*. 2010; Miyata *et al*. 2010; Gruber *et al*. 2011). Furthermore, recent studies indicate that the regulation of cell cycle and growth arrest may play some roles in subsequent differentiation and maturation steps of postmitotic neurons. Neural progenitors exhibit a cell cycle-dependent nuclear movement within the ventricular zone, named interkinetic nuclear migration (INM), which influences cell fate determination as well as neurogenesis, at least in zebrafish retina (Kosodo 2012). In addition, several cell cycle-related proteins have additional functions in the postmitotic neurons of the developing cerebral cortex (Frank & Tsai 2009). For example, the function of p27^kip1^, a regulator for cell cycle exit, switches after growth arrest to regulate the migration and morphology of postmitotic neurons through actin cytoskeletal organization (Kawauchi *et al*. 2006). In mature neurons, the disturbance of growth arrest, which induces cell cycle re-entry, eventually leads to cell death (Herrup & Yang 2007). Thus, growth arrest confers dual functions on cell cycle-related proteins, and disrupting growth arrest may be associated with neurodegenerative diseases. In this review article, we introduce the mechanisms for neurogenesis and neuronal maturation, particularly focusing on INM and neuronal migration, respectively, and discuss the possible roles of growth arrest in brain development and several neurological disorders, such as developmental and neurodegenerative diseases.

## Neural progenitor cells in mammalian cerebral cortex

**Figure 1:**
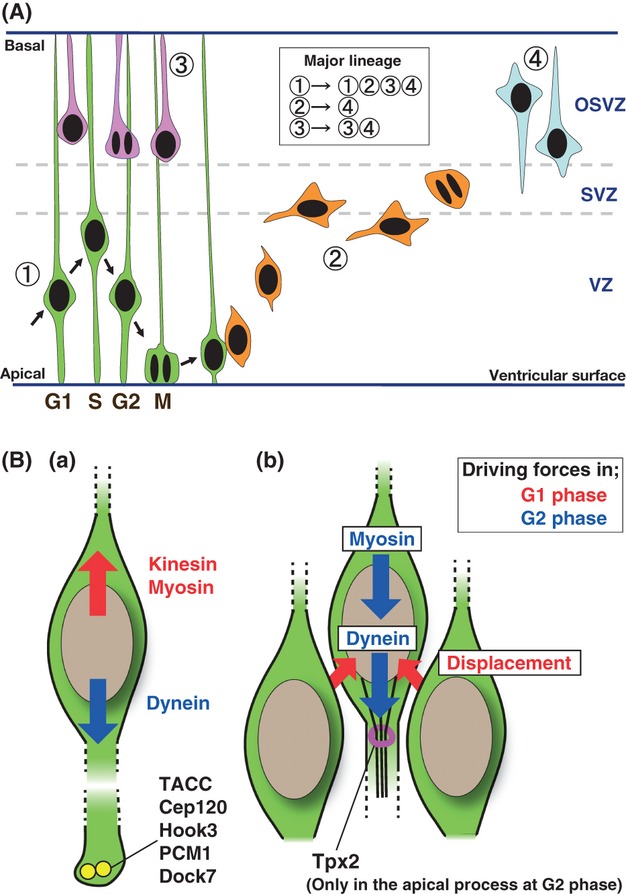
Major lineage of neural progenitors in mammalian cerebral cortex and interkinetic nuclear migration (INM) of apical progenitor. (A) Three kinds of neural progenitors identified in developing mammalian cortex (1–3) and postmitotic neuron (4) are illustrated. 1: Apical progenitor (green). 2: Basal progenitor (orange). 3: OSVZ progenitor (magenta). 4: Postmitotic neuron (light blue). Reported representative lineages from each progenitor (Fietz & Huttner 2011; Lui *et al*. 2011) are indicated in the square box. Cell cycle phases (G1, S, G2 and M) and the nuclear movement in each phase (arrow) of apical progenitor are described. VZ, ventricular zone; SVZ, subventricular zone; and OSVZ, outer subventricular zone. See text for details. (B) Schematics of mode of nuclear movements and proposed driving forces for INM. Arrows show directions of nuclear movements in each cell cycle phase (red: G1 phase, blue: G2 phase). Proposed driving forces for each direction of nuclear movement are indicated as (a) two opposing driving forces, (b) uni-directed driving force and displacement effect for the other direction from surrounded nuclei. See text for detail. The centrosome (yellow) may play an important role in INM because the functions of many centrosomal proteins are involved in INM. Tpx2 protein (magenta) is required for INM (basal-to-apical movement) and only observed in the apical process at G2 phase during interphase, suggesting that Tpx2 links cell cycle machinery with INM. See text for detail.

Neural progenitor cells, opposed to their offspring, postmitotic neurons, exhibit cell cycle progression and cell division during brain development. Before the onset of neurogenesis, neural progenitor cells expand their numbers by symmetric, proliferative division, that is, one progenitor cell produces two progenitor cells (also called ‘self-renewal division’). After neurogenesis begins, the division mode switches to asymmetric division, that is, one progenitor cell produces one progenitor and one neuron or other type of progenitor (Gotz & Huttner 2005; Fietz & Huttner 2011). Currently, at least three types of neural progenitor cells have been identified in the developing mammalian cerebral cortex (Fig. 1A): apical progenitor, basal progenitor, and outer subventricular zone (OSVZ) progenitor (Fietz & Huttner 2011; Lui *et al*. 2011). An apical progenitor [also known as a neuroepithelial cell or radial glial cell (Gotz & Huttner 2005)] is an epithelial cell possessing two long processes along its apico-basal polarity and undergoes both symmetric, proliferative division and asymmetric, neurogenic division at the most apical end (ventricular side) of the ventricular zone (VZ) (Fig. 1A, green). A basal progenitor [also known as an intermediate progenitor (Noctor *et al*. 2004) or nonsurface dividing cell (Miyata *et al*. 2004)] lacks obvious processes and undergoes mostly symmetric, neurogenic division at the basal end of the VZ and subventricular zone (SVZ) (Fig. 1A, orange). An OSVZ progenitor [also known as an outer radial glial (oRG) cell (Hansen *et al*. 2010)] undergoes asymmetric, neurogenic division at the OSVZ, the inner region of brain parenchyma that is partitioned from the SVZ in primate cortex (Smart *et al*. 2002) (Fig. 1A, magenta). Notably, time-lapse lineage analyses have showed that apical progenitors can produce all three types of progenitor cells, but other types of progenitors do not produce the apical progenitors (Miyata *et al*. 2001, 2004; Noctor *et al*. 2001, 2004; Haubensak *et al*. 2004; Shitamukai *et al*. 2011; Wang *et al*. 2011) (Fig. 1A). Thus, apical progenitors can be considered as the stem of all neural progenitor subtypes.

## Interkinetic nuclear migration

### What is INM?

During the progression of cell cycle phases, the nucleus of an apical progenitor conducts a unique mode of movement, named as ‘INM’ or ‘elevator movement’ (Fig. 1). INM is initially proposed by Sauer in 1935 in the embryonic vertebrate neural tube (Sauer 1935). Sauer postulated that the translocation of nuclear position occurs in accordance with the cell cycle progression; the cell division (M phase) of the neural progenitor cells takes place at the apical (ventricular) surface, followed by the nuclear movement from apical to basal during G1 phase. S phase occurs at the most basal end of the VZ and then the nucleus comes back to the apical position in G2 phase for the next cell division. A couple of decades after the first report, an experimental proof of the concept was demonstrated by labeling S-phase nuclei with ^3^H-thymidine, resulting in the appearance of radioactive label–incorporated chromatids in M-phase cells at the apical surface (Sauer & Walker 1959; Sidman *et al*. 1959; Fujita 1962). Recent advances in both light microscopy and tissue culturing methods (Miyata *et al*. 2001; Noctor *et al*. 2001) allow direct time-lapse imaging of INM.

INM has been identified not only in the embryonic neural tube of vertebrates, but also in other pseudostratified epithelial systems including invertebrates. For instance, retina in the developmental stage is a good model to analyze INM because of its relatively simple structure and accessibility for various experimental approaches, especially live-imaging to track nuclear migrations (Baye & Link 2007; Agathocleous & Harris 2009). Although much knowledge about INM has been derived from studies in the central nervous system (ectodermal origin), it has been demonstrated that INM also occurs in endoderm-originated digestive organs such as epithelia emanating from the liver bud (Bort *et al*. 2006) or intestin (Grosse *et al*. 2011) during development. Considering the evolutional aspect, it is important to compare vertebrate and invertebrate systems to clarify the types of molecules originally used for INM. Recent studies demonstrating the existence of INM in the *Drosophila* wing disc (Meyer *et al*. 2011) and *Nematostella* ectoderm (Meyer *et al*. 2011; Nakanishi *et al*. 2012) showed that both microtubule and actomyosin motor systems (see below) are required in more phylogenetically primitive organisms, suggesting that it is difficult to presume which motor system was primarily acquired during the nervous system evolution (Kosodo 2012).

### Molecular mechanisms of INM

It has been a fascinating trial to uncover the mechanism of INM; how does the direction of nuclear migration correlate to each phase of cell cycle? Using drug treatments to disrupt cellular cytoskeletons, the importance of actin (Messier & Auclair 1974; Murciano *et al*. 2002) and microtubule (Langman *et al*. 1966; Karfunkel 1972) organization for INM was determined. Moreover, the molecular machineries controlling several steps of INM were recently revealed by advanced genetic manipulations. For the basal-to-apical nuclear migration, the association of the dynein motor proteins with Lis1 to the microtubule cytoskeleton plays a major role (Gambello *et al*. 2003; Tsai *et al*. 2005). Dynactin-1 and NudC, proteins forming a complex with dynein/Lis1, are also required for the basal-to-apical nuclear migration (Del Bene *et al*. 2008; Cappello *et al*. 2011). Centrosomes, which localize at the apical surface during interphase (Chenn *et al*. 1998), act as a microtubule-organizing center. The disruption of centrosomal protein functions, such as TACC, Cep120, Hook3, PCM1, and Dock7, have been found to perturb INM progression (Xie *et al*. 2007; Ge *et al*. 2010; Yang *et al*. 2012) (Fig. 1B). KASH proteins and SUN proteins form a physical link between the nuclear envelope and the dynein complex (Del Bene *et al*. 2008; Zhang *et al*. 2009; Yu *et al*. 2011). In spite of accumulated evidence that the microtubule motor system is important for the basal-to-apical nuclear migration, this is not always the case in INM of all epithelial tissue. It has been reported that in the zebrafish retina (Norden *et al*. 2009; Leung *et al*. 2011) and *Drosophila* wing disc (Meyer *et al*. 2011), not the dynein/microtubule motor system but the nonmuscle myosin with actin cytoskeleton is the main driver for the basal-to-apical nuclear migration. Interestingly, Rac1, a Rho family small GTPase involved in both microtubule and actin cytoskeletal regulation (Kawauchi 2011), is reported to control the basal-to-apical nuclear migration of neural progenitors (Minobe *et al*. 2009).

In contrast to what is known basal-to-apical nuclear migration, little information is available for apical-to-basal migration. Recent studies propose significant roles for kinesin, microtubule-associated motor, or actomyosin systems in nuclear apical-to-basal movement (Schenk *et al*. 2009; Tsai *et al*. 2010) (Fig. 1Ba). Notably, a critical role for physical displacement as a nonautonomous driving force of INM has been independently demonstrated in two systems (Fig. 1Bb). In developing zebrafish retina, it has been implicated that the trajectories of nuclear movements are largely stochastic, as mathematically postulated to fit nuclear positions (Norden *et al*. 2009). Subsequently, development of time-lapse quantitative analysis of nuclear movement in retina and hindbrain of zebrafish led to the conclusion that stochastic nuclear movement during phases other than the G2 phases arises passively in response to apical migration in neighboring cells (Leung *et al*. 2011). In developing mouse cortex, it was demonstrated that apical-to-basal migration is driven by a crowding effect in the epithelial tissue that results from continuous accumulation of nuclei due to the basal-to-apical active nuclear migration. This conclusion is achieved by nonautonomous movement of fluorescent beads from apical to basal, perturbation of basally oriented movement by disruption of basal-to-apical movement of surrounding cells, and simulation analysis (Kosodo *et al*. 2011). For active basal-to-apical movement, the actomyosin (Norden *et al*. 2009) or dynein/microtubule (Kosodo *et al*. 2011) motor system is used (Fig. 1Bb). The uni-directed active movement in INM would help to minimize the imbalance of nuclear density in the apical and basal regions of pseudostratified epithelia so as to preserve the homeostasis of tissue architecture during these developmental stages (Kosodo *et al*. 2011).

## Cell cycle regulations associated with INM

### Relationship between cell cycle regulation and INM

As discussed in the previous section, the nuclear movement in INM is tightly coupled to the cell cycle progression. From this standpoint, it arises the following questions: whether cell cycle progression can be a driver of INM or whether the nuclear positions can control the cell cycle progression? Inhibition of INM by the chemical inhibitor-mediated disruption of microtubule or actomyosin has been shown to have essentially no effect on cell cycle progression (Karfunkel 1972; Messier & Auclair 1974; Messier 1978; Gambello *et al*. 2003). However, treatment with drugs that interfere with several cell cycle steps result in the ectopic accumulation of nuclei in the neuroepithelia of developing mouse and zebrafish (Ueno *et al*. 2006; Kosodo *et al*. 2011; Leung *et al*. 2011). At a molecular resolution, G1 phase arrest, achieved by overexpressing p18^Ink4c^, an inhibitor protein of cyclin-dependent kinase (CDK) 4 and/or CDK6 (Sherr & Roberts 1999; Thullberg *et al*. 2000), leads to the accumulation of nuclei at a basal position in the VZ of developing mouse brains (Kosodo *et al*. 2011). Taken together, these results indicate that cell cycle progression likely regulates the activity of migration machineries.

How then, does the cell cycle progression correlate to the driving force of INM? It has been demonstrated that the function of Tpx2 protein connects cell cycle phases to the organization of the microtubule cytoskeleton required for INM. Tpx2, a microtubule-associated protein, is not observed in G1 phase, but appears during S phase and accumulates during G2 phase and then strongly associates to the mitotic spindle in M phase in HeLa cells (Gruss *et al*. 2002). In the apical progenitors in mouse brains, Tpx2 localizes on the microtubule in the apical process (but not in the basal process) of G2-phase cells, but not in G1 phase (Kosodo *et al*. 2011). Microtubule bundles in the apical processes of G2 phase are loosened by knockdown of Tpx2, resulting in a perturbation of basal-to-apical nuclear migration (Kosodo *et al*. 2011). Another study reported cell cycle control of actomyosin motor systems in zebrafish retina. Visualization of myosin regulatory light chain tagged with fluorescent protein showed its G2 specific recruitment to the basal side of nuclei. This is required for the basal-to-apical nuclear migration, likely by squeezing nuclei toward the apical side of the neuroepithelium (Leung *et al*. 2011).

### Possible involvement of INM in fate determination

As described above, our understanding of INM has greatly expanded, especially with regard to the molecular machineries that generate the forces of nuclear migrations. What remains to be uncovered in the next stage of research is clarification as to whether INM is linked to cell fate determination, particularly in the developing central nervous system (Kosodo 2012). Interestingly, certain correlations between the S-phase positions and the cell fate of neural stem cell exist. In the retina of zebrafish, proliferative cells can be distinguished from neurogenic cells as different populations by the distance of S-phase positions from the apical surface (Baye & Link 2007). One possible scenario to generate this difference in cell fate is the concentration gradient of morphogen or signaling molecule along the axis of apico-basal polarity within the tissue, with nuclei receiving different amounts of neurogenic factor at specific cell cycle phases during INM (Latasa *et al*. 2009). In support of this hypothesis, Notch signaling–related proteins, whose activity can promote proliferation and cell cycle re-entry of neural stem cells (Pierfelice *et al*. 2011), show heterogenous apico-basal distributions. INM defects caused by a dynactin mutation result in altered exposure to Notch signals and impair neurogenesis in zebrafish retina (Del Bene *et al*. 2008).

Provided that S-phase positioning is one of the regulating factors of cell fate in apical progenitors, it is important to consider where nuclei enter into S phase. Using an elegant time-lapse study in the developing zebrafish nervous system, nuclear movement in each stage of cell cycle has been described (Leung *et al*. 2011); there is a basal drift at the beginning of G1 phase, strong basal-to-apical movement in G2 phase, and complete stochastic movement during S phase. This result essentially matches the nuclear movements observed in the developing mouse cortex (Kosodo *et al*. 2011).

Given that S-phase nuclei have no underlying directionality, how are the positions of S phase determined? Here, we need to consider the length of G1 phase and the mechanism of apical-to-basal nuclear migration during G1 phase (see previous section). If apical-to-basal nuclear movement is driven by an active motor system, it is likely that the position at the end of G1 phase (just before S-phase entry) from the apical surface toward the basal region changes in proportion to the length of G1 phase. However, if G1 nuclear movement is driven by a passive displacement factor, the position of the S-phase cell is likely to be dependent on both the length of G1 phase and the proportion of G2-phase length to the entire cell cycle. A recent report on accelerating the G1 phase of neural progenitors in the developing mouse brain may answer this question.

Co-over-expression of Cdk4 and cyclinD1 using *in utero* electroporation in the developing mouse cortex results in a shortened G1 phase, which evokes delayed neurogenesis (Lange *et al*. 2009). In this study, INM progression with over-expression or down-regulation of Cdk4/cyclinD1, which causes shortening or lengthening of G1 phase, respectively, is examined. Surprisingly, the positions of S-phase entry and exit are essentially the same between untransfected cells and electroporated cells in both shortened and lengthened G1 phase without affecting the number of apical progenitors (Lange *et al*. 2009). The experimental results show that the length of G1 phase was shortened to 65% by the over-expression of Cdk4 and cyclinD1 (from 9.0 to 5.9 h). As the position of S-phase entry is same in the overexpressed situation, this data do not appear to fit the active migration model unless the velocity of G1-phase nuclei was increased due to a side effect of Cdk4 and cyclinD1 over-expression on the motor system for the apical-to-basal nuclear migration. Next, it was demonstrated that the proportion of G2 phase (including M phase) to the entire cell cycle length increases by 1.36 times (from 14% to 19%) in the Cdk4 and cyclinD1 over-expressed condition. An increased proportion in the G2 phase raises the number of descending nuclei in a unit of time, which results in the higher density of nuclei in the apical region. According to the displacement model (see previous section), increased density of the apical region would raise the pressure to translocate nuclei in G1 phase from apical to basal. This might increase the velocity of apical-to-basal nuclear migration and compensate for a shortened G1-phase length, which would result in no obvious change for the nuclear position of S-phase entry. Perhaps, such a robust mechanism of INM might minimize effects of local disturbances of cell cycle progression on the architecture of the developing brain.

## Neuronal migration

### Multistep mode of neuronal migration

**Figure 2:**
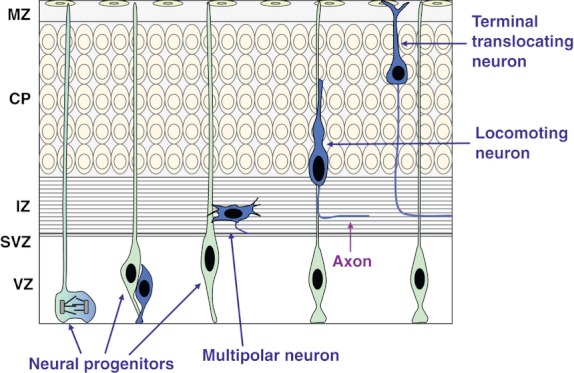
Multistep mode of neuronal migration. Postmitotic excitatory neurons are generated at the ventricular zone (VZ) or subventricular zone (SVZ) (See the enlarged drawing of the VZ and SVZ in Fig. 1) and migrate radially toward the pial surface (Blue cells). Neurons first display multipolar morphology at the lower part of the intermediate zone (IZ) and transform into locomoting neurons. Locomoting neurons possess a leading process and migrate over a long distance along radial glial fibers with elongation of an axon in a reverse direction. The migration mode switches from the locomotion mode into a radial glial fiber-independent terminal translocation mode during the final phase of migration. CP, cortical plate; IZ, intermediate zone; MZ, marginal zone; SVZ, subventricular zone; and VZ, ventricular zone.

Newly generated immature neurons begin the pial surface-directed migration from the ventricular (apical) side, which is essential for the formation of architectural and functional cerebral cortex with a six-layered structure (Rakic 2006; Ayala *et al*. 2007; Kawauchi & Hoshino 2008; Marin *et al*. 2010; Govek *et al*. 2011; Kwan *et al*. 2012). A number of previous studies have indicated that migrating neurons exhibit multistep migration with various morphological changes (Kawauchi & Hoshino 2008) (Fig. 2). Migrating neurons first exhibit multipolar morphologies and subsequently form a leading process and an axon while retracting other neurites (Stensaas 1967; Shoukimas & Hinds 1978; Tamamaki *et al*. 2001; Tabata & Nakajima 2003; Noctor *et al*. 2004). The resulting bipolar-shaped neurons, called locomoting neurons, migrate over long distances along radial glial fibers, apical progenitor-derived long processes, with backward elongation of their axons (locomotion mode) (Rakic 1972, 2006; Nadarajah *et al*. 2001; Hatanaka & Murakami 2002; Noctor *et al*. 2004). At the final phase of migration, neurons switch from the migration mode into a radial glial fiber-independent terminal translocation mode (Nadarajah *et al*. 2001; Sekine *et al*. 2011). During the terminal translocation, dendrite maturation begins. Thus, neuronal migration is required for not only finding the final position but also neuronal maturation (Fig. 2). Defects in neuronal migration cause several neurological disorders, such as periventricular heterotopia and lissencephaly (Gleeson & Walsh 2000; Kawauchi & Hoshino 2008).

### c-jun N-terminal kinase pathway and microtubule-associated proteins

**Figure 3:**
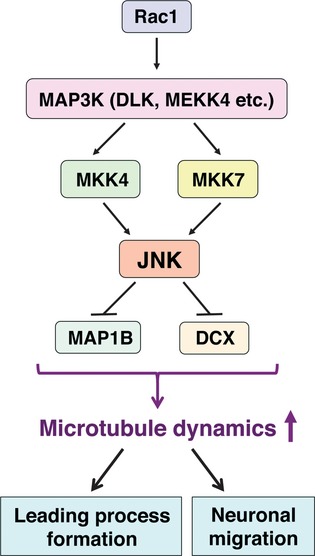
c-jun N-terminal kinase (JNK) pathway in postmitotic migrating neurons. JNK is required for the formation of a leading process (a surface-directed thick neurite of a locomoting neuron, see Fig. 2) and neuronal migration through the regulation of microtubule dynamics. MAP1B and DCX stabilize microtubules, but the phosphorylation by JNK enhances their dissociation from microtubules, resulting in an increase in microtubule dynamics.

The first molecules identified to be involved in the morphological changes of migrating immature neurons were a Rho family small GTPase, Rac1, and its downstream kinase, c-jun N-terminal kinase (JNK) (Kawauchi *et al*. 2003) (Fig. 3). JNK regulates the transition from multipolar cells into locomoting neurons. JNK phosphorylates several microtubule-associated proteins, such as microtubule-associated protein 1B (MAP1B) and DCX (also known as doublecortin) (Chang *et al*. 2003; Kawauchi *et al*. 2003, 2005; Gdalyahu *et al*. 2004) (Fig. 3). Mutations in *DCX* gene cause X-linked lissencephaly in males and subcortical band heterotopia (also known as double cortex syndrome) in females (Gleeson *et al*. 1998; des Portes *et al*. 1998). Although both MAP1B and DCX promote microtubule stability (Francis *et al*. 1999; Gleeson *et al*. 1999; Goold *et al*. 1999; Horesh *et al*. 1999; Gordon-Weeks & Fischer 2000; Kawauchi *et al*. 2005; Trivedi *et al*. 2005), JNK-mediated phosphorylation diminishes their microtubule-binding affinities, resulting in decreased the microtubule stability (that is, increases the microtubule dynamics) (Chang *et al*. 2003; Kawauchi *et al*. 2003, 2005; Gdalyahu *et al*. 2004). Consistent with the fact that microtubule stability is kept at low levels at the tips of neurites (Shea 1999), phosphorylated MAP1B is strongly observed at the tips of axons (Goold *et al*. 1999; Gordon-Weeks & Fischer 2000). It has been reported that suppression of JNK or MAP1B disturbs neurite elongation (Takei *et al*. 2000; Kawauchi *et al*. 2003; Oliva *et al*. 2006; Eto *et al*. 2010). *In vivo* suppression of JNK disturbs the leading process morphology of migrating neurons and the pial surface-directed neuronal migration (Kawauchi *et al*. 2003).

As JNK belongs to a MAP kinase family, its activity is controlled by MAPKKs and MAPKKKs (Huang *et al*. 2004) (Fig. 3). Gene disruption of MKK4 or MKK7, MAPKKs for JNK, delays neuronal migration and disturbs axon formation (Wang *et al*. 2007; Yamasaki *et al*. 2011). Although the phosphorylation of MAP1B, but not DCX, is decreased in MKK4-deficient mice, the phosphorylation of both is suppressed in the MKK7 knockout mice. In addition, inhibition of DLK/MUK, a MAPKKK for JNK, results in similar phenotypes (Hirai *et al*. 2006). Interestingly, gene targeting for MEKK4, another MAPKKK for JNK, shows severe migration defects, resembling periventricular heterotopia (Sarkisian *et al*. 2006). Filamin A, a causative gene product of periventricular heterotopia (Fox *et al*. 1998), has also been reported to mediate the JNK signaling pathway in non-neuronal cells (Nomachi *et al*. 2008; Nakagawa *et al*. 2010) as well as the morphological changes and migration of cortical neurons (Nagano *et al*. 2004). Thus, the JNK-mediated pathway has important roles in neuronal migration and axon formation, and its defects may be associated with several cortical malformations.

### Cdk5 and cell adhesion

**Figure 4:**
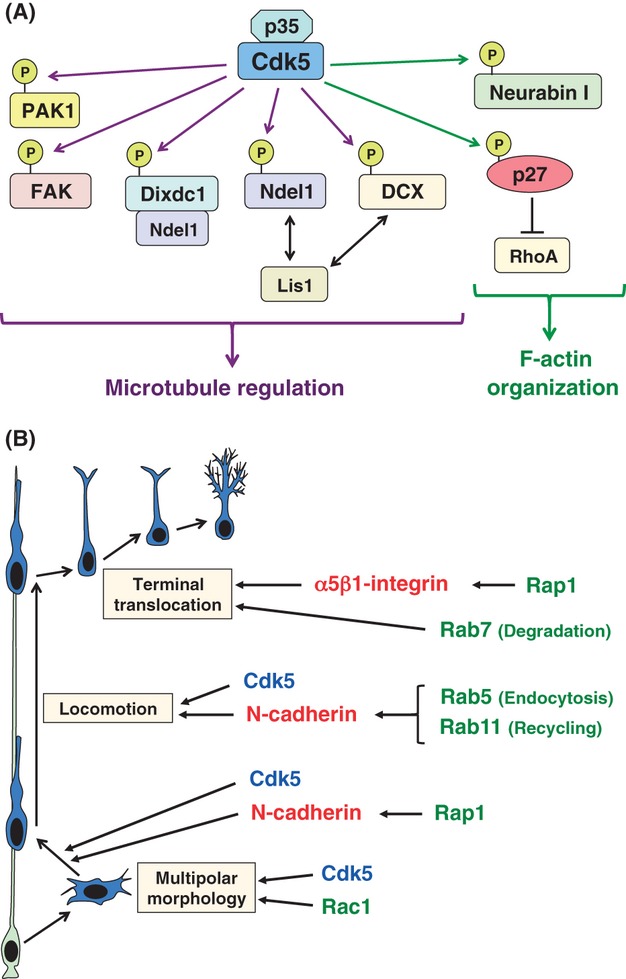
Roles of Cdk5 and cell adhesion molecules in multistep mode of neuronal migration. (A) Cdk5 phosphorylates many substrate molecules, including microtubule- and actin cytoskeleton-regulatory proteins (purple and green arrows, respectively). (B) Cdk5 is required for multiple steps of neuronal migration. Cdk5 (blue) regulates multipolar morphology of migrating neurons in a p27^kip1^-dependent manner, but its function in the transition into locomoting neurons is independent of p27^kip1^ as suppression of p27^kip1^ does not affect this step. Several small GTPases (green) also play important roles in the multistep mode of neuronal migration. Their functions are partly mediated by the regulation of cell adhesion molecules, N-cadherin and α5β1-integrin (red).

DCX is also phosphorylated by cyclin-dependent kinase 5 (Cdk5) and MAP/microtubule affinity–regulating kinase 2 (MARK2, also known as Par-1) (Schaar *et al*. 2004; Tanaka *et al*. 2004) (Fig. 4A). Cdk5 is an unconventional CDK because its activity is mainly observed in postmitotic neurons (Tsai *et al*. 1993). Cdk5 is activated by p35, p39, and cyclin I, but not cyclin D, E, and A (Lee *et al*. 1996; Hisanaga & Saito 2003; Brinkkoetter *et al*. 2009; Su & Tsai 2011). *In vivo* suppression of Cdk5 activity by gene targeting, *in vivo* RNA interference and dominant negative experiments, has been shown to lead to severe neuronal migration defects (Ohshima *et al*. 1996; Gilmore *et al*. 1998; Kawauchi *et al*. 2003, 2006) (Fig. 4B). Similar to JNK, Cdk5 is required for the formation of leading process of migrating immature neurons (Kawauchi *et al*. 2006). However, Cdk5 also regulates multipolar cell morphologies, compared to the lesser effect of JNK on this aspect (Hirai *et al*. 2006; Kawauchi *et al*. 2006). A recent study showed that Cdk5 activity is required for the locomotion mode of neuronal migration (Nishimura *et al*. 2010), indicating that Cdk5 is a central regulator for multistep migration of immature neurons (Fig. 4B).

Cdk5 phosphorylates many substrate molecules, including p27^kip1^ (Kawauchi *et al*. 2006), Dixdc1 (Singh *et al*. 2010), Ndel1 (also known as Nudel) (Niethammer *et al*. 2000), focal adhesion kinase (FAK) (Xie *et al*. 2003), p21-activated kinase 1 (PAK1) (Rashid *et al*. 2001), neurabin I (Causeret *et al*. 2007), as well as DCX (Tanaka *et al*. 2004) (Fig. 4A). Ndel1 binds to Lis1, a causative gene product for lissencephaly (Reiner *et al*. 1993), and Ndel1 and Lis1 cooperatively control cytoplasmic dynein functions (Niethammer *et al*. 2000; Sasaki *et al*. 2000; Yamada *et al*. 2008). The Ndel1 phosphorylated by Cdk5 interacts with 14-3-3ε, which regulates the localization of Ndel1 and Lis1 (Toyo-oka *et al*. 2003). FAK is phosphorylated on Ser732 by Cdk5, and this phosphorylation is required for perinuclear microtubule organization (Xie *et al*. 2003). However, Cdk5 phosphorylates a neuron-specific F-actin-binding protein, neurabin I (Causeret *et al*. 2007). Furthermore, Cdk5-mediated phosphorylation of p27^kip1^ promotes actin reorganization, as described below. *In vivo* suppression of these Cdk5 substrates, p27^kip1^, Ndel1, FAK, and Neurabin I, disturbs neuronal migration mainly due to cytoskeletal defects.

In addition to cytoskeletal proteins, Cdk5 is known to regulate cell adhesion. Cell adhesion can be classified into cell-to-cell adhesion and cell-to-extracellular matrix (ECM) adhesion (Kawauchi 2012). Recent studies indicate that N-cadherin-mediated cell-to-cell adhesion plays essential roles in the multipolar and locomotion modes of neuronal migration (Kawauchi *et al*. 2010; Shikanai *et al*. 2011), whereas α5β1-integrin, a cell-to-ECM adhesion molecule that binds to fibronectin (Kawauchi 2012), is required for the terminal translocation (Sekine *et al*. 2012) (Fig. 4B). Rab family small GTPases, Rab5 and Rab11, regulate the intracellular trafficking of N-cadherin, which is required for the locomotion mode of neuronal migration (Kawauchi *et al*. 2010; Kawauchi 2011). A ras family small GTPase, Rap1, promotes the activities of N-cadherin and integrin at the early and final phases of neuronal migration, respectively (Franco *et al*. 2011; Jossin & Cooper 2011; Sekine *et al*. 2012) (Fig. 4B). Interestingly, Cdk5 can control both N-cadherin and integrin in a small GTPase-independent manner *in vitro* (Kwon *et al*. 2000; Huang *et al*. 2009), although it is still unclear whether Cdk5-mediated regulation of cell adhesion is involved in neuronal migration *in vivo*.

## Linking mechanisms of cell cycle exit and neuronal migration

### Cdk5 and p27^kip1^ in cell cycle exit, neuronal differentiation and migration

The cell cycle exit, neuronal differentiation, and migration occur concurrently, along with suppression in the activities of cyclin–CDKs. However, as described above, Cdk5 is strongly activated in postmitotic neurons. Although many studies indicate that Cdk5 is a regulator for cytoskeletal organization and signal transduction, rather than cell cycle, some notable facts remain. One is that Cdk5 directly phosphorylates p27^kip1^, a CDK inhibitor protein (Kawauchi *et al*. 2006). In addition, some mature neurons in the cortical plate abnormally re-enter the cell cycle in Cdk5-deficient mice (Cicero & Herrup 2005), similar to what is observed in the brains of p27^kip1^/p19^Ink4d^ double knockout mice (Zindy *et al*. 1999), suggesting a functional relationship between Cdk5 and other cell cycle proteins.

**Figure 5:**
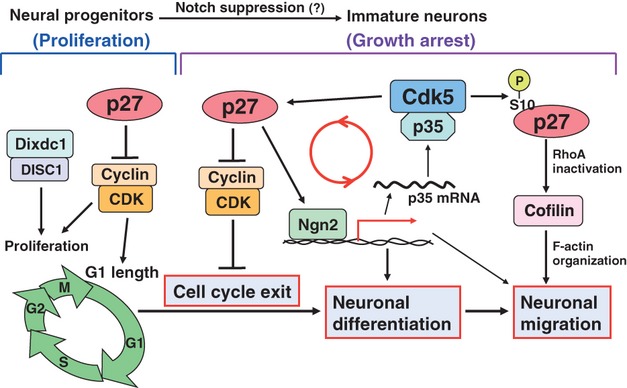
A possible link in mechanisms between cell cycle exit, neuronal differentiation, and neuronal migration. In the developing cerebral cortex, cell cycle exit, neuronal differentiation, and initiation of neuronal migration occur concurrently. A cyclin-dependent kinase (CDK) inhibitor protein, p27^kip1^, controls the G1 length and cell cycle exit in neural progenitors via the suppression of Cyclin-CDK activities. In addition to these cell cycle regulatory functions, p27^kip1^ promotes neuronal differentiation via the up-regulation of Ngn2 protein level and neuronal migration through the suppression of RhoA activity and thereby activation of an actin-binding protein, Cofilin. Ngn2 activates the transcription of *p35* as well as neuronal differentiation-related genes. In postmitotic neurons, p35 binds to and activates Cdk5, which directly phosphorylates and stabilizes p27^kip1^ protein and is required for the maintenance of growth arrest. A proposed feedback loop of Cdk5/p35-p27^kip1^-Ngn2-p35-Cdk5 is shown (red circle).

It is known that p27^kip1^ regulates G1 length and cell cycle exit in the ventricular zone of the developing cerebral cortex via suppression of conventional CDK activities (Sherr & Roberts 1999; Mitsuhashi *et al*. 2001; Tarui *et al*. 2005). In contrast, Ser10 of p27^kip1^ is phosphorylated by Cdk5 in postmitotic neurons and this phosphorylation promotes its protein stability through the protection of p27^kip1^ from proteasome-dependent protein degradation (Ishida *et al*. 2000; Kotake *et al*. 2005; Kawauchi *et al*. 2006), suggesting that Cdk5 is an upstream positive regulator for p27^kip1^, a CDK inhibitor protein, in G0-arrested neurons, although p27^kip1^ acts as a negative regulator for conventional CDKs (Fig. 5). Furthermore, the increased protein levels of p27^kip1^ have essential roles in cortical neuronal migration and the formation of multipolar cell morphologies (Kawauchi *et al*. 2006). Cdk5-p27^kip1^ pathway enhances actin reorganization via the suppression of RhoA activity and thereby activation of an actin-binding protein, cofilin (Kawauchi *et al*. 2006). It has been reported that p27^kip1^ is also involved in the regulation of microtubule organization (Baldassarre *et al*. 2005; Godin *et al*. 2012). Interestingly, a recent study indicates that connexin 43, a component of gap junction involved in both neural progenitor proliferation and neuronal migration (Elias & Kriegstein 2008), acts upstream of p27^kip1^ to regulate the multipolar morphology of migrating neurons (Liu *et al*. 2012). Taken together, these findings suggest that p27^kip1^ acquires additional functions in cytoskeletal regulation and neuronal migration during growth arrest and that this functional switch is mediated at least in part by Cdk5 (Fig. 5).

Cdk5-mediated phosphorylation of Dixdc1 also functions as a molecular switch between neural progenitor proliferation and neuronal migration (Singh *et al*. 2010). Nonphosphorylated Dixdc1 binds to Disrupted in Schizophrenia-1 (DISC1) and controls neural progenitor proliferation. In contrast, Cdk5 phosphorylates Dixdc1 in postmitotic neurons, resulting in increased interaction between Ndel1 and DISC1 and promotion of neuronal migration.

In addition to the dual functions in neural progenitors and migrating neurons, p27^kip1^ is involved in neuronal differentiation. A previous report showed that p27^kip1^ increases the protein levels of Neurogenin 2 (Ngn2), a basic helix-loop-helix-type transcription factor required for neuronal differentiation, and promotes neuronal differentiation (Nguyen *et al*. 2006). Furthermore, Cdk5 deficiency partially disturbs neuronal differentiation (Cicero & Herrup 2005; Zheng *et al*. 2010) as well as neuronal migration, and Cdk5-mediated phosphorylation of p27^kip1^ at Ser10 and Thr187 is involved in the regulation of neuronal differentiation (Zheng *et al*. 2010). Interestingly, p35, an activator for Cdk5, was identified as a target molecule of Ngn2 (Ge *et al*. 2006), and it has been reported that Ngn2 is also required for neuronal migration (Hand *et al*. 2005; Ge *et al*. 2006; Heng *et al*. 2008). These findings implicate a positive feedback loop of Cdk5/p35-p27^kip1^-Ngn2-p35 that has important roles in the growth arrest–associated neuronal differentiation and initiation of migration (Kawauchi & Hoshino 2008) (Fig. 5). The identity of the molecule(s) that turn on the positive feedback loop for the synchronized cellular events of cell cycle exit, neuronal differentiation, and initiation of neuronal migration is still unclear, but there is evidence to indicate that Notch signaling suppresses p27^kip1^ mRNA and/or protein levels (Sarmento *et al*. 2005; Vernon *et al*. 2006; Murata *et al*. 2009), suggesting that weakened Notch signal may enhance p27^kip1^ expression and thereby the positive feedback loop.

### Other CDK inhibitor proteins and Rb-E2F

**Figure 6:**
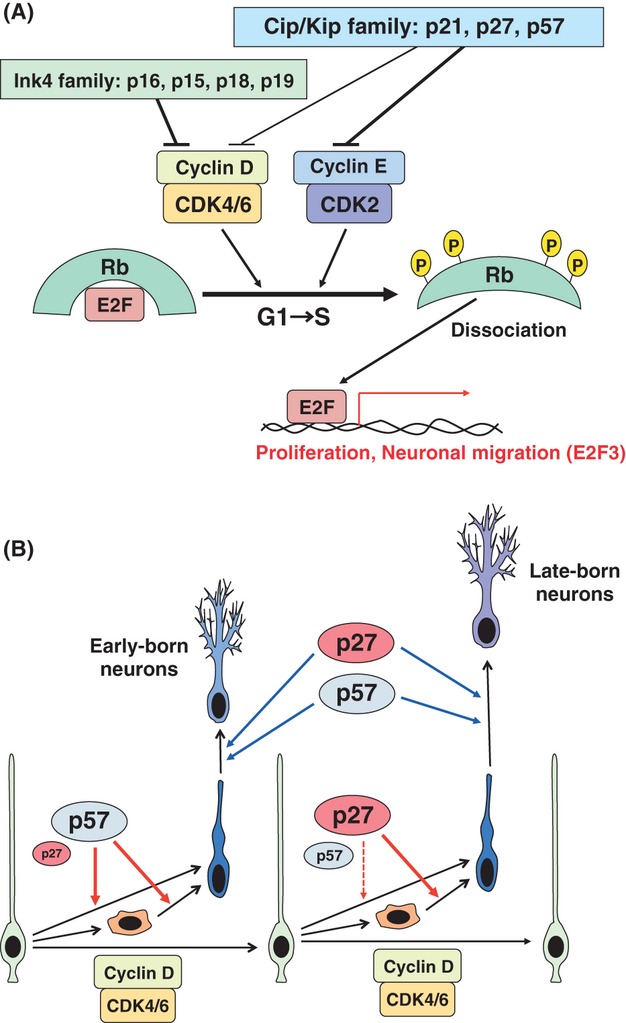
Cyclin-dependent kinase (CDK) inhibitor proteins regulate cell cycle progression, growth arrest, and postmitotic neuronal migration. (A) Molecular mechanisms for G1/S transition. The transition from G1 to S phase is dependent on CyclinD-Cdk4/6 and CyclinE-Cdk2 activities, which phosphorylate Rb protein. The phosphorylated Rb protein dissociates E2F family transcription factors. Both E2F1 and E2F3 promote G1/S transition in neural progenitors, whereas E2F3, but not E2F1, regulates neuronal positioning. The activities of Cyclin-CDK complexes are suppressed by CDK inhibitor proteins, which are composed of a Cip/Kip family (p21^cip1^, p27^kip1^, and p57^kip2^) and Ink4 family (p16^Ink4a^, p15^Ink4b^, p18^Ink4c^, and p19^Ink4d^). (B) Roles of CDK inhibitor proteins, p27^kip1^ and p57^kip2^, in cell cycle exit and subsequent neuronal migration. p57^kip2^ and p27^kip1^ preferentially control the cell cycle exit of neural progenitors for early-born (deep layer) and late-born (upper layer) neurons, respectively. p27^kip1^ mainly functions in basal progenitors (orange cells) rather than apical progenitors (green cells). Both p27^kip1^ and p57^kip2^ have been shown to regulate the migration of postmitotic neurons as well as the cell cycle exit.

Other cell cycle-related proteins have also been reported to have dual functions in proliferating and arrested cells. CDK inhibitor proteins include members of Cip/Kip (p21^cip1^, p27^kip1^, and p57^kip2^) and Ink4 (p16^Ink4a^, p15^Ink4b^, p18^Ink4c^, and p19^Ink4d^) families (Sherr & Roberts 1999) (Fig. 6A). Although p57^kip2^ mainly controls the cell cycle exit of early-born neurons (deep layer neurons), p27^kip1^ preferentially regulates the growth arrest of late-born neurons (upper layer neurons) (Mairet-Coello *et al*. 2012) (Fig. 6B). In the postmitotic neurons, it has been reported that not only p27^kip1^ but also p57^kip2^ is involved in neuronal migration (Itoh *et al*. 2007). Consistently, both proteins are localized at the leading process and cell soma as well as nucleus in migrating neurons (Kawauchi *et al*. 2006).

Furthermore, retinoblastoma (Rb) protein and E2F family transcription factors are reported to regulate both cell cycle in neural progenitors and migration in postmitotic neurons. Rb protein binds to and represses the E2F functions, whereas Cdk-dependent phosphorylation of Rb dissociates E2Fs from the Rb protein, allowing E2Fs to interact with target DNA sequences (Giacinti & Giordano 2006) (Fig. 6A). Knockout of the *Rb* gene perturbs the neuronal positioning in cerebral cortex, and the phenotypes are rescued by double knockout of Rb and E2F3, but not E2F1 (Ferguson *et al*. 2005; McClellan *et al*. 2007). Although the switching mechanism of Rb-E2F functions is unclear, a recent study shows that Cdk5 has the ability to phosphorylate Rb protein (Futatsugi *et al*. 2012). In addition to the regulators for G1/S transition, Aurora A and anaphase-promoting complex/cyclosome (APC/C), both of which mainly function at M phase, are reported to regulate neuronal migration and axon/dendrite formation (Konishi *et al*. 2004; Kim *et al*. 2009; Mori *et al*. 2009; Takitoh *et al*. 2012). Therefore, growth arrest signals may provide additional functions beyond cell cycle regulation for some cell cycle-related proteins.

## Growth arrest and developmental neurological disorders

Disruption of the balance between progenitor self-renewal and cell cycle exit (neuronal differentiation) leads to several neurological disorders. For example, abnormally enhanced cell cycle exit of neural progenitors leads to premature differentiation and thereby exhaustion of neural progenitors, resulting in microcephaly (small brain) (Mochida & Walsh 2004; Bond & Woods 2006; Lizarraga *et al*. 2010; Miyata *et al*. 2010; Buchman *et al*. 2011; Gruber *et al*. 2011). Interestingly, microcephaly is sometimes accompanied by neuronal migration disorders. Mutation in *ArfGEF2* causes microcephaly and periventricular heterotopia (Sheen *et al*. 2004). *ArfGEF2* encodes Big2/ArfGEF2 protein, which regulates membrane trafficking from Golgi apparatus via the activation of Arf family small GTPases. Furthermore, it is reported that Big2 is also localized at recycling endosomes (Shin *et al*. 2004). Consistent with this, endocytosis and recycling of a cell-cell adhesion molecule, N-cadherin, are known to play essential roles in the locomotion mode of neuronal migration (Kawauchi *et al*. 2010; Shikanai *et al*. 2011). Interestingly, N-cadherin is also required for the maintenance of neuroepithelial (ventricular zone) structures (Kadowaki *et al*. 2007), whose disruption is observed in the brains with periventricular heterotopia (Ferland *et al*. 2009). Therefore, the regulation of membrane trafficking may be another mechanism that links neural progenitor proliferation and neuronal migration.

Human mutations in the *Nde1* gene result in microcephaly with lissencephaly (referred to as ‘microlissencephaly’) (Feng & Walsh 2004; Alkuraya *et al*. 2011). Furthermore, knockdown of *abnormal spindle microcephaly* (*ASPM*), a causative gene for autosomal recessive primary microcephaly (MCPH, for microcephaly primary hereditary), disturbs neuronal migration as well as neural progenitor proliferation in mice (Fish *et al*. 2006; Buchman *et al*. 2011). In addition to human neurological disorder–related genes, many molecules, including Lis1, dynein, SUN proteins, and Rac1, are required for both INM and neuronal migration (Hirotsune *et al*. 1998; Gambello *et al*. 2003; Kawauchi *et al*. 2003; Tsai *et al*. 2005, 2007; Yoshizawa *et al*. 2005; Minobe *et al*. 2009; Zhang *et al*. 2009; Kawauchi 2011; Yu *et al*. 2011). Because most of these proteins function in both neural progenitors and postmitotic neurons, neural progenitor proliferation and neuronal migration share several common intracellular pathways in centrosome and/or microtubule regulation. Considering that Cdk5 acts upstream of Lis1, dynein, and Rac1 (Niethammer *et al*. 2000; Xin *et al*. 2004; Govek *et al*. 2011) and that p27^kip1^ is involved in the regulation of microtubules as well as actin cytoskeleton (Baldassarre *et al*. 2005; Kawauchi *et al*. 2006; Godin *et al*. 2012), the growth arrest-mediated Cdk5 activation by the up-regulation of p35 protein may alter the function of several cell cycle-related proteins, which exert different cellular events in part using common machineries.

## Growth arrest in postmitotic mature cells

In adulthood, many cells, including mature neurons, maintain a quiescent state throughout life. It has been reported that cyclin E binds to and suppresses the activity of Cdk5, resulting in the enhancement of synapse formation (Odajima *et al*. 2011). This suggests that some cell cycle-related proteins also function in mature neurons. Thus, alternative functions for cell cycle-related proteins are important for growth-arrested cells. However, several studies have indicated that cell cycle re-entry by perturbing growth arrest is a trigger for cell death.

**Figure 7:**
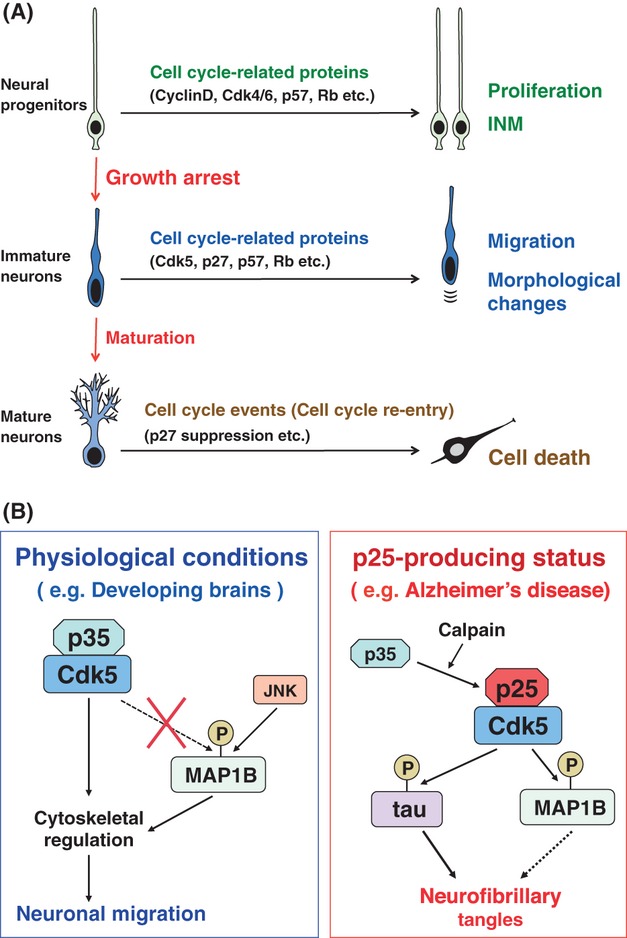
Alternative functions of cell cycle-related proteins in the construction and maintenance of brains throughout life. (A) Cell cycle-related proteins function in not only the proliferation of neural progenitors but also various aspects of brain construction and its maintenance throughout life. Cell cycle machinery controls interkinetic nuclear migration (INM) in neural progenitors, and after growth arrest, several cell cycle-related proteins change their functions to control the migration and morphology of postmitotic neurons. However, cell cycle re-entry by disturbance of growth arrest is thought to trigger cell death. (B) Cdk5 functions in brain development and neurodegenerative diseases. Cdk5, binding to its activator, p35, phosphorylates many substrate molecules and controls the multistep mode of neuronal migration in developing brains (see Fig. 4). In contrast, p35 is cleaved into the more stable p25 in pathogenic conditions, including Alzheimer's disease. Cdk5/p25, but not Cdk5/p35, strongly phosphorylates tau and MAP1B, which may be associated with the formation of neurofibrillary tangles in neurodegenerative diseased brains.

Mammalian auditory epithelium, composed of hair cells and supporting cells, has limited capability for regeneration, which remains an obstacle for the development of therapeutics for sensorineural hearing loss (Roberson & Rubel 1994; Forge *et al*. 1998; White *et al*. 2006). In contrast, in the avian auditory epithelium, the loss of hair cells leads to re-entry of supporting cells into the cell cycle, giving rise to both hair cells and supporting cells (Corwin & Cotanche 1988; Ryals & Rubel 1988). For the purpose of promoting regeneration of the cochlea in mammals, knockdown of p27^kip1^ in the postmitotic supporting cells of mouse auditory epithelia was performed (Ono *et al*. 2009). That study reported the successful re-activation of the proliferative capacities of the auditory supporting cells, but induction of the apoptotic pathway occurred several days later (Fig. 7A).

Re-activation of cell cycle machinery in mature neurons is also associated with cell death. In the brains of Alzheimer's disease mouse models, re-expression of cell cycle proteins, such as cyclin A and PCNA, and DNA replication are observed before neuronal cell death (Yang *et al*. 2001, 2003; Varvel *et al*. 2008). These ‘cell cycle events’ themselves do not seem to directly induce neuronal cell death, but are thought to be important priming phenomena for neurodegenerative diseases (Yang & Herrup 2007). Furthermore, it has been reported that the abnormal activation of Cdk5 is involved in neurodegeneration. Inhibition of Cdk5 induces cell cycle events, suggesting that Cdk5 suppresses the cell cycle in mature neurons (Cicero & Herrup 2005; Zhang *et al*. 2008). The activator for Cdk5 is changed from p35 into a more stable isoform, p25, through a calpain-mediated cleavage in brains with neurodegenerative diseases (Patrick *et al*. 1999; Kusakawa *et al*. 2000; Lee *et al*. 2000). It is known that Cdk5/p35 and Cdk5/p25 exhibit different substrate specificities. Unlike Cdk5/p35, Cdk5/p25 strongly phosphorylates tau and MAP1B (Patrick *et al*. 1999; Kawauchi *et al*. 2005), and their hyperphosphorylation is observed in Alzheimer's diseased brains (Hasegawa *et al*. 1990; Ulloa *et al*. 1994; Cruz *et al*. 2003; Hisanaga & Saito 2003; Tsai *et al*. 2004; Su & Tsai 2011). Cdk5/p25 interacts with and inhibits the activity of histone deacetylase 1 (HDAC1), and suppression of HDAC1 induces double-stranded DNA breaks and cell cycle activity in neurons (Kim *et al*. 2008). These results indicate that the re-activation of cell cycle machinery, including DNA replication, in mature postmitotic cells induces cell death and further suggest that the growth arrest of mature neurons plays essential roles in neuronal survival and normal brain functions.

## Conclusion remarks

The tight regulation of cell cycle proteins is essential for the proliferation and cell cycle exit of neural progenitors during brain development. Recent studies also indicate that cell cycle-related proteins contribute to much broader events beyond the cell cycle regulation in the developing and adult brains (Fig. 7A). In neural progenitors, the cell cycle machinery is closely associated with and actively controls INM at least in part through Tpx2-mediated organization of microtubules. Even after growth arrest, cell cycle-related proteins, such as p27^kip1^ and Rb, exhibit alternative functions that affect the migration and changes in morphology of postmitotic neurons. Interestingly, although these alternative functions are essential for brain development, disruption of growth arrest in mature neurons or other postmitotic cells is closely associated with cell death, suggesting that re-activation of cell cycle progression itself may be harmful to postmitotic neurons. As a large proportion of cells in adulthood are in a postmitotic state, it is possible that growth arrest contributes to the maintenance of cellular homeostasis in the whole body.

## References

[b1] Agathocleous M, Harris WA (2009). From progenitors to differentiated cells in the vertebrate retina. Annu. Rev. Cell Dev. Biol.

[b2] Alkuraya FS, Cai X, Emery C, Mochida GH, Al-Dosari MS, Felie JM, Hill RS, Barry BJ, Partlow JN, Gascon GG, Kentab A, Jan M, Shaheen R, Feng Y, Walsh CA (2011). Human mutations in NDE1 cause extreme microcephaly with lissencephaly. Am. J. Hum. Genet.

[b3] Ayala R, Shu T, Tsai LH (2007). Trekking across the brain: the journey of neuronal migration. Cell.

[b4] Baldassarre G, Belletti B, Nicoloso MS, Schiappacassi M, Vecchione A, Spessotto P, Morrione A, Canzonieri V, Colombatti A (2005). p27(Kip1)-stathmin interaction influences sarcoma cell migration and invasion. Cancer Cell.

[b5] Baye LM, Link BA (2007). Interkinetic nuclear migration and the selection of neurogenic cell divisions during vertebrate retinogenesis. J. Neurosci.

[b6] Bond J, Woods CG (2006). Cytoskeletal genes regulating brain size. Curr. Opin. Cell Biol.

[b7] Bort R, Signore M, Tremblay K, Martinez Barbera JP, Zaret KS (2006). Hex homeobox gene controls the transition of the endoderm to a pseudostratified, cell emergent epithelium for liver bud development. Dev. Biol.

[b8] Brinkkoetter PT, Olivier P, Wu JS, Henderson S, Krofft RD, Pippin JW, Hockenbery D, Roberts JM, Shankland SJ (2009). Cyclin I activates Cdk5 and regulates expression of Bcl-2 and Bcl-XL in postmitotic mouse cells. J. Clin. Invest.

[b9] Buchman JJ, Durak O, Tsai LH (2011). ASPM regulates Wnt signaling pathway activity in the developing brain. Genes Dev.

[b10] Cappello S, Monzo P, Vallee RB (2011). NudC is required for interkinetic nuclear migration and neuronal migration during neocortical development. Dev. Biol.

[b11] Causeret F, Jacobs T, Terao M, Heath O, Hoshino M, Nikolic M (2007). Neurabin-I is phosphorylated by Cdk5: implications for neuronal morphogenesis and cortical migration. Mol. Biol. Cell.

[b12] Chang L, Jones Y, Ellisman MH, Goldstein LS, Karin M (2003). JNK1 is required for maintenance of neuronal microtubules and controls phosphorylation of microtubule-associated proteins. Dev. Cell.

[b13] Chenn A, Zhang YA, Chang BT, McConnell SK (1998). Intrinsic polarity of mammalian neuroepithelial cells. Mol. Cell. Neurosci.

[b14] Cicero S, Herrup K (2005). Cyclin-dependent kinase 5 is essential for neuronal cell cycle arrest and differentiation. J. Neurosci.

[b15] Corwin JT, Cotanche DA (1988). Regeneration of sensory hair cells after acoustic trauma. Science.

[b16] Cruz JC, Tseng HC, Goldman JA, Shih H, Tsai LH (2003). Aberrant Cdk5 activation by p25 triggers pathological events leading to neurodegeneration and neurofibrillary tangles. Neuron.

[b17] Del Bene F, Wehman AM, Link BA, Baier H (2008). Regulation of neurogenesis by interkinetic nuclear migration through an apical-basal notch gradient. Cell.

[b18] Elias LA, Kriegstein AR (2008). Gap junctions: multifaceted regulators of embryonic cortical development. Trends Neurosci.

[b19] Eto K, Kawauchi T, Osawa M, Tabata H, Nakajima K (2010). Role of dual leucine zipper-bearing kinase (DLK/MUK/ZPK) in axonal growth. Neurosci. Res.

[b20] Feng Y, Walsh CA (2004). Mitotic spindle regulation by Nde1 controls cerebral cortical size. Neuron.

[b21] Ferguson KL, McClellan KA, Vanderluit JL, McIntosh WC, Schuurmans C, Polleux F, Slack RS (2005). A cell-autonomous requirement for the cell cycle regulatory protein, Rb, in neuronal migration. EMBO J.

[b22] Ferland RJ, Batiz LF, Neal J (2009). Disruption of neural progenitors along the ventricular and subventricular zones in periventricular heterotopia. Hum. Mol. Genet.

[b23] Fietz SA, Huttner WB (2011). Cortical progenitor expansion, self-renewal and neurogenesis-a polarized perspective. Curr. Opin. Neurobiol.

[b24] Fish JL, Kosodo Y, Enard W, Paabo S, Huttner WB (2006). Aspm specifically maintains symmetric proliferative divisions of neuroepithelial cells. Proc. Natl Acad. Sci. USA.

[b25] Forge A, Li L, Nevill G (1998). Hair cell recovery in the vestibular sensory epithelia of mature guinea pigs. J. Comp. Neurol.

[b26] Fox JW, Lamperti ED, Eksioglu YZ, Hong SE, Feng Y, Graham DA, Scheffer IE, Dobyns WB, Hirsch BA, Radtke RA, Berkovic SF, Huttenlocher PR, Walsh CA (1998). Mutations in filamin 1 prevent migration of cerebral cortical neurons in human periventricular heterotopia. Neuron.

[b27] Francis F, Koulakoff A, Boucher D, Chafey P, Schaar B, Vinet MC, Friocourt G, McDonnell N, Reiner O, Kahn A, McConnell SK, Berwald-Netter Y, Denoulet P, Chelly J (1999). Doublecortin is a developmentally regulated, microtubule-associated protein expressed in migrating and differentiating neurons. Neuron.

[b28] Franco SJ, Martinez-Garay I, Gil-Sanz C, Harkins-Perry SR, Muller U (2011). Reelin regulates cadherin function via Dab1/Rap1 to control neuronal migration and lamination in the neocortex. Neuron.

[b29] Frank CL, Tsai LH (2009). Alternative functions of core cell cycle regulators in neuronal migration, neuronal maturation, and synaptic plasticity. Neuron.

[b30] Fujita S (1962). Kinetics of cellular proliferation. Exp. Cell Res.

[b31] Futatsugi A, Utreras E, Rudrabhatla P, Jaffe H, Pant HC, Kulkarni AB (2012). Cyclin-dependent kinase 5 regulates E2F transcription factor through phosphorylation of Rb protein in neurons. Cell Cycle.

[b32] Gambello MJ, Darling DL, Yingling J, Tanaka T, Gleeson JG, Wynshaw-Boris A (2003). Multiple dose-dependent effects of Lis1 on cerebral cortical development. J. Neurosci.

[b33] Gdalyahu A, Ghosh I, Levy T, Sapir T, Sapoznik S, Fishler Y, Azoulai D, Reiner O (2004). DCX, a new mediator of the JNK pathway. EMBO J.

[b34] Ge W, He F, Kim KJ (2006). Coupling of cell migration with neurogenesis by proneural bHLH factors. Proc. Natl Acad. Sci. USA.

[b35] Ge X, Frank CL, Calderon de Anda F, Tsai LH (2010). Hook3 interacts with PCM1 to regulate pericentriolar material assembly and the timing of neurogenesis. Neuron.

[b36] Giacinti C, Giordano A (2006). RB and cell cycle progression. Oncogene.

[b37] Gilmore EC, Ohshima T, Goffinet AM, Kulkarni AB, Herrup K (1998). Cyclin-dependent kinase 5-deficient mice demonstrate novel developmental arrest in cerebral cortex. J. Neurosci.

[b38] Gleeson JG, Allen KM, Fox JW, Lamperti ED, Berkovic S, Scheffer I, Cooper EC, Dobyns WB, Minnerath SR, Ross ME, Walsh CA (1998). Doublecortin, a brain-specific gene mutated in human X-linked lissencephaly and double cortex syndrome, encodes a putative signaling protein. Cell.

[b39] Gleeson JG, Lin PT, Flanagan LA, Walsh CA (1999). Doublecortin is a microtubule-associated protein and is expressed widely by migrating neurons. Neuron.

[b40] Gleeson JG, Walsh CA (2000). Neuronal migration disorders: from genetic diseases to developmental mechanisms. Trends Neurosci.

[b41] Godin JD, Thomas N, Laguesse S (2012). p27(Kip1) is a microtubule-associated protein that promotes microtubule polymerization during neuron migration. Dev. Cell.

[b42] Goold RG, Owen R, Gordon-Weeks PR (1999). Glycogen synthase kinase 3beta phosphorylation of microtubule-associated protein 1B regulates the stability of microtubules in growth cones. J. Cell Sci.

[b43] Gordon-Weeks PR, Fischer I (2000). MAP1B expression and microtubule stability in growing and regenerating axons. Microsc. Res. Tech.

[b44] Gotz M, Huttner WB (2005). The cell biology of neurogenesis. Nat. Rev. Mol. Cell Biol.

[b45] Govek EE, Hatten ME, Van Aelst L (2011). The role of Rho GTPase proteins in CNS neuronal migration. Dev. Neurobiol.

[b46] Grosse AS, Pressprich MF, Curley LB, Hamilton KL, Margolis B, Hildebrand JD, Gumucio DL (2011). Cell dynamics in fetal intestinal epithelium: implications for intestinal growth and morphogenesis. Development.

[b47] Gruber R, Zhou Z, Sukchev M, Joerss T, Frappart PO, Wang ZQ (2011). MCPH1 regulates the neuroprogenitor division mode by coupling the centrosomal cycle with mitotic entry through the Chk1-Cdc25 pathway. Nat. Cell Biol.

[b48] Gruss OJ, Wittmann M, Yokoyama H, Pepperkok R, Kufer T, Sillje H, Karsenti E, Mattaj IW, Vernos I (2002). Chromosome-induced microtubule assembly mediated by TPX2 is required for spindle formation in HeLa cells. Nat. Cell Biol.

[b49] Hand R, Bortone D, Mattar P, Nguyen L, Heng JI, Guerrier S, Boutt E, Peters E, Barnes AP, Parras C, Schuurmans C, Guillemot F, Polleux F (2005). Phosphorylation of Neurogenin2 specifies the migration properties and the dendritic morphology of pyramidal neurons in the neocortex. Neuron.

[b50] Hansen DV, Lui JH, Parker PR, Kriegstein AR (2010). Neurogenic radial glia in the outer subventricular zone of human neocortex. Nature.

[b51] Hasegawa M, Arai T, Ihara Y (1990). Immunochemical evidence that fragments of phosphorylated MAP5 (MAP1B) are bound to neurofibrillary tangles in Alzheimer's disease. Neuron.

[b52] Hatanaka Y, Murakami F (2002). *In vitro* analysis of the origin, migratory behavior, and maturation of cortical pyramidal cells. J. Comp. Neurol.

[b53] Haubensak W, Attardo A, Denk W, Huttner WB (2004). Neurons arise in the basal neuroepithelium of the early mammalian telencephalon: a major site of neurogenesis. Proc. Natl Acad. Sci. USA.

[b54] Heng JI, Nguyen L, Castro DS, Zimmer C, Wildner H, Armant O, Skowronska-Krawczyk D, Bedogni F, Matter JM, Hevner R, Guillemot F (2008). Neurogenin 2 controls cortical neuron migration through regulation of Rnd2. Nature.

[b55] Herrup K, Yang Y (2007). Cell cycle regulation in the postmitotic neuron: oxymoron or new biology?. Nat. Rev. Neurosci.

[b56] Hirai SI, Feng Cui D, Miyata T, Ogawa M, Kiyonari H, Suda Y, Aizawa S, Banba Y, Ohno S (2006). The c-Jun N-terminal kinase activator dual leucine zipper kinase regulates axon growth and neuronal migration in the developing cerebral cortex. J. Neurosci.

[b57] Hirotsune S, Fleck MW, Gambello MJ, Bix GJ, Chen A, Clark GD, Ledbetter DH, McBain CJ, Wynshaw-Boris A (1998). Graded reduction of Pafah1b1 (Lis1) activity results in neuronal migration defects and early embryonic lethality. Nat. Genet.

[b58] Hisanaga S, Saito T (2003). The regulation of cyclin-dependent kinase 5 activity through the metabolism of p35 or p39 Cdk5 activator. Neurosignals.

[b59] Horesh D, Sapir T, Francis F, Wolf SG, Caspi M, Elbaum M, Chelly J, Reiner O (1999). Doublecortin, a stabilizer of microtubules. Hum. Mol. Genet.

[b60] Huang C, Jacobson K, Schaller MD (2004). MAP kinases and cell migration. J. Cell Sci.

[b61] Huang C, Rajfur Z, Yousefi N, Chen Z, Jacobson K, Ginsberg MH (2009). Talin phosphorylation by Cdk5 regulates Smurf1-mediated talin head ubiquitylation and cell migration. Nat. Cell Biol.

[b62] Ishida N, Kitagawa M, Hatakeyama S, Nakayama K (2000). Phosphorylation at serine 10, a major phosphorylation site of p27(Kip1), increases its protein stability. J. Biol. Chem.

[b63] Itoh Y, Masuyama N, Nakayama K, Nakayama KI, Gotoh Y (2007). The cyclin-dependent kinase inhibitors p57 and p27 regulate neuronal migration in the developing mouse neocortex. J. Biol. Chem.

[b64] Jossin Y, Cooper JA (2011). Reelin, Rap1 and N-cadherin orient the migration of multipolar neurons in the developing neocortex. Nat. Neurosci.

[b65] Kadowaki M, Nakamura S, Machon O, Krauss S, Radice GL, Takeichi M (2007). N-cadherin mediates cortical organization in the mouse brain. Dev. Biol.

[b66] Karfunkel P (1972). The activity of microtubules and microfilaments in neurulation in the chick. J. Exp. Zool.

[b67] Kawauchi T (2011). Regulation of cell adhesion and migration in cortical neurons: not only Rho but also Rab family small GTPases. Small GTPases.

[b68] Kawauchi T (2012). Cell adhesion and its endocytic regulation in cell migration during neural development and cancer metastasis. Int. J. Mol. Sci.

[b69] Kawauchi T, Chihama K, Nabeshima Y, Hoshino M (2003). The *in vivo* roles of STEF/Tiam1, Rac1 and JNK in cortical neuronal migration. EMBO J.

[b70] Kawauchi T, Chihama K, Nabeshima Y, Hoshino M (2006). Cdk5 phosphorylates and stabilizes p27kip1 contributing to actin organization and cortical neuronal migration. Nat. Cell Biol.

[b71] Kawauchi T, Chihama K, Nishimura YV, Nabeshima Y, Hoshino M (2005). MAP1B phosphorylation is differentially regulated by Cdk5/p35, Cdk5/p25, and JNK. Biochem. Biophys. Res. Commun.

[b72] Kawauchi T, Hoshino M (2008). Molecular pathways regulating cytoskeletal organization and morphological changes in migrating neurons. Dev. Neurosci.

[b73] Kawauchi T, Sekine K, Shikanai M, Chihama K, Tomita K, Kubo K, Nakajima K, Nabeshima Y, Hoshino M (2010). Rab GTPases-dependent endocytic pathways regulate neuronal migration and maturation through N-cadherin trafficking. Neuron.

[b74] Kim AH, Puram SV, Bilimoria PM, Ikeuchi Y, Keough S, Wong M, Rowitch D, Bonni A (2009). A centrosomal Cdc20-APC pathway controls dendrite morphogenesis in postmitotic neurons. Cell.

[b75] Kim D, Frank CL, Dobbin MM (2008). Deregulation of HDAC1 by p25/Cdk5 in neurotoxicity. Neuron.

[b76] Konishi Y, Stegmuller J, Matsuda T, Bonni S, Bonni A (2004). Cdh1-APC controls axonal growth and patterning in the mammalian brain. Science.

[b77] Kosodo Y (2012). Interkinetic nuclear migration: beyond a hallmark of neurogenesis. Cell. Mol. Life Sci.

[b78] Kosodo Y, Suetsugu T, Suda M, Mimori-Kiyosue Y, Toida K, Baba SA, Kimura A, Matsuzaki F (2011). Regulation of interkinetic nuclear migration by cell cycle-coupled active and passive mechanisms in the developing brain. EMBO J.

[b79] Kotake Y, Nakayama K, Ishida N, Nakayama KI (2005). Role of serine 10 phosphorylation in p27 stabilization revealed by analysis of p27 knock-in mice harboring a serine 10 mutation. J. Biol. Chem.

[b80] Kusakawa G, Saito T, Onuki R, Ishiguro K, Kishimoto T, Hisanaga S (2000). Calpain-dependent proteolytic cleavage of the p35 cyclin-dependent kinase 5 activator to p25. J. Biol. Chem.

[b81] Kwan KY, Sestan N, Anton ES (2012). Transcriptional co-regulation of neuronal migration and laminar identity in the neocortex. Development.

[b82] Kwon YT, Gupta A, Zhou Y, Nikolic M, Tsai LH (2000). Regulation of N-cadherin-mediated adhesion by the p35-Cdk5 kinase. Curr. Biol.

[b83] Lange C, Huttner WB, Calegari F (2009). Cdk4/cyclinD1 overexpression in neural stem cells shortens G1, delays neurogenesis, and promotes the generation and expansion of basal progenitors. Cell Stem Cell.

[b84] Langman J, Guerrant RL, Freeman BG (1966). Behavior of neuro-epithelial cells during closure of the neural tube. J. Comp. Neurol.

[b85] Latasa MJ, Cisneros E, Frade JM (2009). Cell cycle control of Notch signaling and the functional regionalization of the neuroepithelium during vertebrate neurogenesis. Int. J. Dev. Biol.

[b86] Lee MH, Nikolic M, Baptista CA, Lai E, Tsai LH, Massague J (1996). The brain-specific activator p35 allows Cdk5 to escape inhibition by p27Kip1 in neurons. Proc. Natl Acad. Sci. USA.

[b87] Lee MS, Kwon YT, Li M, Peng J, Friedlander RM, Tsai LH (2000). Neurotoxicity induces cleavage of p35 to p25 by calpain. Nature.

[b88] Leung L, Klopper AV, Grill SW, Harris WA, Norden C (2011). Apical migration of nuclei during G2 is a prerequisite for all nuclear motion in zebrafish neuroepithelia. Development.

[b89] Liu X, Sun L, Torii M, Rakic P (2012). Connexin 43 controls the multipolar phase of neuronal migration to the cerebral cortex. Proc. Natl Acad. Sci. USA.

[b90] Lizarraga SB, Margossian SP, Harris MH, Campagna DR, Han AP, Blevins S, Mudbhary R, Barker JE, Walsh CA, Fleming MD (2010). Cdk5rap2 regulates centrosome function and chromosome segregation in neuronal progenitors. Development.

[b91] Lui JH, Hansen DV, Kriegstein AR (2011). Development and evolution of the human neocortex. Cell.

[b92] Mairet-Coello G, Tury A, Van Buskirk E, Robinson K, Genestine M, DiCicco-Bloom E (2012). p57(KIP2) regulates radial glia and intermediate precursor cell cycle dynamics and lower layer neurogenesis in developing cerebral cortex. Development.

[b93] Marin O, Valiente M, Ge X, Tsai LH (2010). Guiding neuronal cell migrations. Cold Spring Harb. Perspect. Biol.

[b94] McClellan KA, Ruzhynsky VA, Douda DN, Vanderluit JL, Ferguson KL, Chen D, Bremner R, Park DS, Leone G, Slack RS (2007). Unique requirement for Rb/E2F3 in neuronal migration: evidence for cell cycle-independent functions. Mol. Cell. Biol.

[b95] Messier PE (1978). Microtubules, interkinetic nuclear migration and neurulation. Experientia.

[b96] Messier PE, Auclair C (1974). Effect of cytochalasin B on interkinetic nuclear migration in the chick embryo. Dev. Biol.

[b97] Meyer EJ, Ikmi A, Gibson MC (2011). Interkinetic nuclear migration is a broadly conserved feature of cell division in pseudostratified epithelia. Curr. Biol.

[b98] Minobe S, Sakakibara A, Ohdachi T, Kanda R, Kimura M, Nakatani S, Tadokoro R, Ochiai W, Nishizawa Y, Mizoguchi A, Kawauchi T, Miyata T (2009). Rac is involved in the interkinetic nuclear migration of cortical progenitor cells. Neurosci. Res.

[b99] Mitsuhashi T, Aoki Y, Eksioglu YZ, Takahashi T, Bhide PG, Reeves SA, Caviness VS (2001). Overexpression of p27Kip1 lengthens the G1 phase in a mouse model that targets inducible gene expression to central nervous system progenitor cells. Proc. Natl Acad. Sci. USA.

[b100] Miyata T, Kawaguchi A, Okano H, Ogawa M (2001). Asymmetric inheritance of radial glial fibers by cortical neurons. Neuron.

[b101] Miyata T, Kawaguchi A, Saito K, Kawano M, Muto T, Ogawa M (2004). Asymmetric production of surface-dividing and non-surface-dividing cortical progenitor cells. Development.

[b102] Miyata T, Kawaguchi D, Kawaguchi A, Gotoh Y (2010). Mechanisms that regulate the number of neurons during mouse neocortical development. Curr. Opin. Neurobiol.

[b103] Mochida GH, Walsh CA (2004). Genetic basis of developmental malformations of the cerebral cortex. Arch. Neurol.

[b104] Mori D, Yamada M, Mimori-Kiyosue Y, Shirai Y, Suzuki A, Ohno S, Saya H, Wynshaw-Boris A, Hirotsune S (2009). An essential role of the aPKC-Aurora A-NDEL1 pathway in neurite elongation by modulation of microtubule dynamics. Nat. Cell Biol.

[b105] Murata J, Ohtsuka T, Tokunaga A, Nishiike S, Inohara H, Okano H, Kageyama R (2009). Notch-Hes1 pathway contributes to the cochlear prosensory formation potentially through the transcriptional down-regulation of p27Kip1. J. Neurosci. Res.

[b106] Murciano A, Zamora J, Lopez-Sanchez J, Frade JM (2002). Interkinetic nuclear movement may provide spatial clues to the regulation of neurogenesis. Mol. Cell. Neurosci.

[b107] Nadarajah B, Brunstrom JE, Grutzendler J, Wong RO, Pearlman AL (2001). Two modes of radial migration in early development of the cerebral cortex. Nat. Neurosci.

[b108] Nagano T, Morikubo S, Sato M (2004). Filamin A and FILIP (Filamin A-Interacting Protein) regulate cell polarity and motility in neocortical subventricular and intermediate zones during radial migration. J. Neurosci.

[b109] Nakagawa K, Sugahara M, Yamasaki T, Kajiho H, Takahashi S, Hirayama J, Minami Y, Ohta Y, Watanabe T, Hata Y, Katada T, Nishina H (2010). Filamin associates with stress signalling kinases MKK7 and MKK4 and regulates JNK activation. Biochem. J.

[b110] Nakanishi N, Renfer E, Technau U, Rentzsch F (2012). Nervous systems of the sea anemone Nematostella vectensis are generated by ectoderm and endoderm and shaped by distinct mechanisms. Development.

[b111] Nguyen L, Besson A, Heng JI, Schuurmans C, Teboul L, Parras C, Philpott A, Roberts JM, Guillemot F (2006). p27kip1 independently promotes neuronal differentiation and migration in the cerebral cortex. Genes Dev.

[b112] Niethammer M, Smith DS, Ayala R, Peng J, Ko J, Lee MS, Morabito M, Tsai LH (2000). NUDEL is a novel Cdk5 substrate that associates with LIS1 and cytoplasmic dynein. Neuron.

[b113] Nishimura YV, Sekine K, Chihama K, Nakajima K, Hoshino M, Nabeshima Y, Kawauchi T (2010). Dissecting the factors involved in the locomotion mode of neuronal migration in the developing cerebral cortex. J. Biol. Chem.

[b114] Noctor SC, Flint AC, Weissman TA, Dammerman RS, Kriegstein AR (2001). Neurons derived from radial glial cells establish radial units in neocortex. Nature.

[b115] Noctor SC, Martinez-Cerdeno V, Ivic L, Kriegstein AR (2004). Cortical neurons arise in symmetric and asymmetric division zones and migrate through specific phases. Nat. Neurosci.

[b116] Nomachi A, Nishita M, Inaba D, Enomoto M, Hamasaki M, Minami Y (2008). Receptor tyrosine kinase Ror2 mediates Wnt5a-induced polarized cell migration by activating c-Jun N-terminal kinase via actin-binding protein filamin A. J. Biol. Chem.

[b117] Norden C, Young S, Link BA, Harris WA (2009). Actomyosin is the main driver of interkinetic nuclear migration in the retina. Cell.

[b118] Odajima J, Wills ZP, Ndassa YM (2011). Cyclin E constrains Cdk5 activity to regulate synaptic plasticity and memory formation. Dev. Cell.

[b119] Ohshima T, Ward JM, Huh CG, Longenecker G, Veeranna Pant HC, Brady RO, Martin LJ, Kulkarni AB (1996). Targeted disruption of the cyclin-dependent kinase 5 gene results in abnormal corticogenesis, neuronal pathology and perinatal death. Proc. Natl Acad. Sci. USA.

[b120] Oliva AA, Atkins CM, Copenagle L, Banker GA (2006). Activated c-Jun N-terminal kinase is required for axon formation. J. Neurosci.

[b121] Ono K, Nakagawa T, Kojima K, Matsumoto M, Kawauchi T, Hoshino M, Ito J (2009). Silencing p27 reverses post-mitotic state of supporting cells in neonatal mouse cochleae. Mol. Cell. Neurosci.

[b122] Patrick GN, Zukerberg L, de la Nikolic M, Monte S, Dikkes P, Tsai LH (1999). Conversion of p35 to p25 deregulates Cdk5 activity and promotes neurodegeneration. Nature.

[b123] Pierfelice T, Alberi L, Gaiano N (2011). Notch in the vertebrate nervous system: an old dog with new tricks. Neuron.

[b124] des Portes V, Pinard JM, Billuart P, Vinet MC, Koulakoff A, Carrie A, Gelot A, Dupuis E, Motte J, Berwald-Netter Y, Catala M, Kahn A, Beldjord C, Chelly J (1998). A novel CNS gene required for neuronal migration and involved in X-linked subcortical laminar heterotopia and lissencephaly syndrome. Cell.

[b125] Rakic P (1972). Mode of cell migration to the superficial layers of fetal monkey neocortex. J. Comp. Neurol.

[b126] Rakic P (2006). A century of progress in corticoneurogenesis: from silver impregnation to genetic engineering. Cereb. Cortex.

[b127] Rashid T, Banerjee M, Nikolic M (2001). Phosphorylation of Pak1 by the p35/Cdk5 kinase affects neuronal morphology. J. Biol. Chem.

[b128] Reiner O, Carrozzo R, Shen Y, Wehnert M, Faustinella F, Dobyns WB, Caskey CT, Ledbetter DH (1993). Isolation of a Miller-Dieker lissencephaly gene containing G protein beta-subunit-like repeats. Nature.

[b129] Roberson DW, Rubel EW (1994). Cell division in the gerbil cochlea after acoustic trauma. Am. J. Otol.

[b130] Ryals BM, Rubel EW (1988). Hair cell regeneration after acoustic trauma in adult Coturnix quail. Science.

[b131] Sarkisian MR, Bartley CM, Chi H, Nakamura F, Hashimoto-Torii K, Torii M, Flavell RA, Rakic P (2006). MEKK4 signaling regulates filamin expression and neuronal migration. Neuron.

[b132] Sarmento LM, Huang H, Limon A, Gordon W, Fernandes J, Tavares MJ, Miele L, Cardoso AA, Classon M, Carlesso N (2005). Notch1 modulates timing of G1-S progression by inducing SKP2 transcription and p27 Kip1 degradation. J. Exp. Med.

[b133] Sasaki S, Shionoya A, Ishida M, Gambello MJ, Yingling J, Wynshaw-Boris A, Hirotsune S (2000). A LIS1/NUDEL/cytoplasmic dynein heavy chain complex in the developing and adult nervous system. Neuron.

[b134] Sauer FC (1935). Mitosis in the neural tube. J. Comp. Neurol.

[b135] Sauer ME, Walker BE (1959). Radioautographic study of interkinetic nuclear migration in the neural tube. Proc. Soc. Exp. Biol. Med.

[b136] Schaar BT, Kinoshita K, McConnell SK (2004). Doublecortin microtubule affinity is regulated by a balance of kinase and phosphatase activity at the leading edge of migrating neurons. Neuron.

[b137] Schenk J, Wilsch-Brauninger M, Calegari F, Huttner WB (2009). Myosin II is required for interkinetic nuclear migration of neural progenitors. Proc. Natl Acad. Sci. USA.

[b138] Sekine K, Honda T, Kawauchi T, Kubo K, Nakajima K (2011). The outermost region of the developing cortical plate is crucial for both the switch of the radial migration mode and the Dab1-dependent “inside-out” lamination in the neocortex. J. Neurosci.

[b139] Sekine K, Kawauchi T, Kubo K, Honda T, Herz J, Hattori M, Kinashi T, Nakajima K (2012). Reelin controls neuronal positioning by promoting cell-matrix adhesion via inside-out activation of integrin alpha5beta1. Neuron.

[b140] Shea TB (1999). Selective stabilization of microtubules within the proximal region of developing axonal neurites. Brain Res. Bull.

[b141] Sheen VL, Ganesh VS, Topcu M, Sebire G, Bodell A, Hill RS, Grant PE, Shugart YY, Imitola J, Khoury SJ, Guerrini R, Walsh CA (2004). Mutations in ARFGEF2 implicate vesicle trafficking in neural progenitor proliferation and migration in the human cerebral cortex. Nat. Genet.

[b142] Sherr CJ, Roberts JM (1999). CDK inhibitors: positive and negative regulators of G1-phase progression. Genes Dev.

[b143] Shikanai M, Nakajima K, Kawauchi T (2011). N-cadherin regulates radial glial fiber-dependent migration of cortical locomoting neurons. Commun. Integr. Biol.

[b144] Shin HW, Morinaga N, Noda M, Nakayama K (2004). BIG2, a guanine nucleotide exchange factor for ADP-ribosylation factors: its localization to recycling endosomes and implication in the endosome integrity. Mol. Biol. Cell.

[b145] Shitamukai A, Konno D, Matsuzaki F (2011). Oblique radial glial divisions in the developing mouse neocortex induce self-renewing progenitors outside the germinal zone that resemble primate outer subventricular zone progenitors. J. Neurosci.

[b146] Shoukimas GM, Hinds JW (1978). The development of the cerebral cortex in the embryonic mouse: an electron microscopic serial section analysis. J. Comp. Neurol.

[b147] Sidman RL, Miale IL, Feder N (1959). Cell proliferation and migration in the primitive ependymal zone: an autoradiographic study of histogenesis in the nervous system. Exp. Neurol.

[b148] Singh KK, Ge X, Mao Y, Drane L, Meletis K, Samuels BA, Tsai LH (2010). Dixdc1 is a critical regulator of DISC1 and embryonic cortical development. Neuron.

[b149] Smart IH, Dehay C, Giroud P, Berland M, Kennedy H (2002). Unique morphological features of the proliferative zones and postmitotic compartments of the neural epithelium giving rise to striate and extrastriate cortex in the monkey. Cereb. Cortex.

[b150] Stensaas LJ (1967). The development of hippocampal and dorsolateral pallial regions of the cerebral hemisphere in fetal rabbits. II. Twenty millimeter stage, neuroblast morphology. J. Comp. Neurol.

[b151] Su SC, Tsai LH (2011). Cyclin-dependent kinases in brain development and disease. Annu. Rev. Cell Dev. Biol.

[b152] Tabata H, Nakajima K (2003). Multipolar migration: the third mode of radial neuronal migration in the developing cerebral cortex. J. Neurosci.

[b153] Takei Y, Teng J, Harada A, Hirokawa N (2000). Defects in axonal elongation and neuronal migration in mice with disrupted tau and map1b genes. J. Cell Biol.

[b154] Takitoh T, Kumamoto K, Wang CC, Sato M, Toba S, Wynshaw-Boris A, Hirotsune S (2012). Activation of Aurora-A is essential for neuronal migration via modulation of microtubule organization. J. Neurosci.

[b155] Tamamaki N, Nakamura K, Okamoto K, Kaneko T (2001). Radial glia is a progenitor of neocortical neurons in the developing cerebral cortex. Neurosci. Res.

[b156] Tanaka T, Serneo FF, Tseng HC, Kulkarni AB, Tsai LH, Gleeson JG (2004). Cdk5 phosphorylation of doublecortin ser297 regulates its effect on neuronal migration. Neuron.

[b157] Tarui T, Takahashi T, Nowakowski RS, Hayes NL, Bhide PG, Caviness VS (2005). Overexpression of p27 Kip 1, probability of cell cycle exit, and laminar destination of neocortical neurons. Cereb. Cortex.

[b158] Thullberg M, Bartkova J, Khan S, Hansen K, Ronnstrand L, Lukas J, Strauss M, Bartek J (2000). Distinct versus redundant properties among members of the INK4 family of cyclin-dependent kinase inhibitors. FEBS Lett.

[b159] Toyo-oka K, Shionoya A, Gambello MJ, Cardoso C, Leventer R, Ward HL, Ayala R, Tsai LH, Dobyns W, Ledbetter D, Hirotsune S, Wynshaw-Boris A (2003). 14–3-3epsilon is important for neuronal migration by binding to NUDEL: a molecular explanation for Miller-Dieker syndrome. Nat. Genet.

[b160] Trivedi N, Marsh P, Goold RG, Wood-Kaczmar A, Gordon-Weeks PR (2005). Glycogen synthase kinase-3beta phosphorylation of MAP1B at Ser1260 and Thr1265 is spatially restricted to growing axons. J. Cell Sci.

[b161] Tsai JW, Bremner KH, Vallee RB (2007). Dual subcellular roles for LIS1 and dynein in radial neuronal migration in live brain tissue. Nat. Neurosci.

[b162] Tsai JW, Chen Y, Kriegstein AR, Vallee RB (2005). LIS1 RNA interference blocks neural stem cell division, morphogenesis, and motility at multiple stages. J. Cell Biol.

[b163] Tsai JW, Lian WN, Kemal S, Kriegstein AR, Vallee RB (2010). Kinesin 3 and cytoplasmic dynein mediate interkinetic nuclear migration in neural stem cells. Nat. Neurosci.

[b164] Tsai LH, Lee MS, Cruz J (2004). Cdk5, a therapeutic target for Alzheimer's disease?. Biochim. Biophys. Acta.

[b165] Tsai LH, Takahashi T, Caviness VS, Harlow E (1993). Activity and expression pattern of cyclin-dependent kinase 5 in the embryonic mouse nervous system. Development.

[b166] Ueno M, Katayama K, Yamauchi H, Nakayama H, Doi K (2006). Cell cycle progression is required for nuclear migration of neural progenitor cells. Brain Res.

[b167] Ulloa L, Montejo de Garcini E, Gomez-Ramos P, Moran MA, Avila J (1994). Microtubule-associated protein MAP1B showing a fetal phosphorylation pattern is present in sites of neurofibrillary degeneration in brains of Alzheimer's disease patients. Brain Res. Mol. Brain Res.

[b168] Varvel NH, Bhaskar K, Patil AR, Pimplikar SW, Herrup K, Lamb BT (2008). Abeta oligomers induce neuronal cell cycle events in Alzheimer's disease. J. Neurosci.

[b169] Vernon AE, Movassagh M, Horan I, Wise H, Ohnuma S, Philpott A (2006). Notch targets the Cdk inhibitor Xic1 to regulate differentiation but not the cell cycle in neurons. EMBO Rep.

[b170] Wang X, Nadarajah B, Robinson AC, McColl BW, Jin JW, Dajas-Bailador F, Boot-Handford RP, Tournier C (2007). Targeted deletion of the mitogen-activated protein kinase kinase 4 gene in the nervous system causes severe brain developmental defects and premature death. Mol. Cell. Biol.

[b171] Wang X, Tsai JW, LaMonica B, Kriegstein AR (2011). A new subtype of progenitor cell in the mouse embryonic neocortex. Nat. Neurosci.

[b172] White PM, Doetzlhofer A, Lee YS, Groves AK, Segil N (2006). Mammalian cochlear supporting cells can divide and trans-differentiate into hair cells. Nature.

[b173] Xie Z, Moy LY, Sanada K, Zhou Y, Buchman JJ, Tsai LH (2007). Cep120 and TACCs control interkinetic nuclear migration and the neural progenitor pool. Neuron.

[b174] Xie Z, Sanada K, Samuels BA, Shih H, Tsai LH (2003). Serine 732 phosphorylation of FAK by Cdk5 is important for microtubule organization, nuclear movement, and neuronal migration. Cell.

[b175] Xin X, Ferraro F, Back N, Eipper BA, Mains RE (2004). Cdk5 and Trio modulate endocrine cell exocytosis. J. Cell Sci.

[b176] Yamada M, Toba S, Yoshida Y, Haratani K, Mori D, Yano Y, Mimori-Kiyosue Y, Nakamura T, Itoh K, Fushiki S, Setou M, Wynshaw-Boris A, Torisawa T, Toyoshima YY, Hirotsune S (2008). LIS1 and NDEL1 coordinate the plus-end-directed transport of cytoplasmic dynein. EMBO J.

[b177] Yamasaki T, Kawasaki H, Arakawa S, Shimizu K, Shimizu S, Reiner O, Okano H, Nishina S, Azuma N, Penninger JM, Katada T, Nishina H (2011). Stress-activated protein kinase MKK7 regulates axon elongation in the developing cerebral cortex. J. Neurosci.

[b178] Yang Y, Geldmacher DS, Herrup K (2001). DNA replication precedes neuronal cell death in Alzheimer's disease. J. Neurosci.

[b179] Yang Y, Herrup K (2007). Cell division in the CNS: protective response or lethal event in post-mitotic neurons?. Biochim. Biophys. Acta.

[b180] Yang Y, Mufson EJ, Herrup K (2003). Neuronal cell death is preceded by cell cycle events at all stages of Alzheimer's disease. J. Neurosci.

[b181] Yang YT, Wang CL, Van Aelst L (2012). DOCK7 interacts with TACC3 to regulate interkinetic nuclear migration and cortical neurogenesis. Nat. Neurosci.

[b182] Yoshizawa M, Kawauchi T, Sone M, Nishimura YV, Terao M, Chihama K, Nabeshima Y, Hoshino M (2005). Involvement of a Rac activator, P-Rex1, in neurotrophin-derived signaling and neuronal migration. J. Neurosci.

[b183] Yu J, Lei K, Zhou M, Craft CM, Xu G, Xu T, Zhuang Y, Xu R, Han M (2011). KASH protein Syne-2/Nesprin-2 and SUN proteins SUN1/2 mediate nuclear migration during mammalian retinal development. Hum. Mol. Genet.

[b184] Zhang J, Cicero SA, Wang L, Romito-Digiacomo RR, Yang Y, Herrup K (2008). Nuclear localization of Cdk5 is a key determinant in the postmitotic state of neurons. Proc. Natl Acad. Sci. USA.

[b185] Zhang X, Lei K, Yuan X, Wu X, Zhuang Y, Xu T, Xu R, Han M (2009). SUN1/2 and Syne/Nesprin-1/2 complexes connect centrosome to the nucleus during neurogenesis and neuronal migration in mice. Neuron.

[b186] Zheng YL, Li BS, Rudrabhatla P, Shukla V, Amin ND, Maric D, Kesavapany S, Kanungo J, Pareek TK, Takahashi S, Grant P, Kulkarni AB, Pant HC (2010). Phosphorylation of p27Kip1 at Thr187 by cyclin-dependent kinase 5 modulates neural stem cell differentiation. Mol. Biol. Cell.

[b187] Zindy F, Cunningham JJ, Sherr CJ, Jogal S, Smeyne RJ, Roussel MF (1999). Postnatal neuronal proliferation in mice lacking Ink4d and Kip1 inhibitors of cyclin-dependent kinases. Proc. Natl Acad. Sci. USA.

